# Imidazole Alkaloids from the South China Sea Sponge *Pericharax heteroraphis* and Their Cytotoxic and Antiviral Activities

**DOI:** 10.3390/molecules21020150

**Published:** 2016-01-26

**Authors:** Kai-Kai Gong, Xu-Li Tang, Yi-Sheng Liu, Ping-Lin Li, Guo-Qiang Li

**Affiliations:** 1 Department of Pharmacy, Binzhou Medical University Hospital, Yellow River Second Road 661, Binzhou 256603, China; gongkaikai1005@163.com (K.-K.G.); byfy_lys@163.com (Y.-S.L.); 2 Key Laboratory of Marine Drugs, Chinese Ministry of Education, School of Medicine and Pharmacy, Ocean University of China, Yushan Road 5, Qingdao 266003, China; 3 College of Chemistry and Chemical Engineering, Ocean University of China, Songling Road 238, Qingdao 266100, China; tangxuli@ouc.edu.cn

**Keywords:** sponge, *Pericharax heteroraphis*, 2-aminoimidazole alkaloids, cytotoxicities, anti-H1N1 activity

## Abstract

Marine sponges continue to serve as a rich source of alkaloids possessing interesting biological activities and often exhibiting unique structural frameworks. In the current study, chemical investigation on the marine sponge *Pericharax heteroraphis* collected from the South China Sea yielded one new imidazole alkaloid named naamidine J (**1**) along with four known ones (**2**–**5**). Their structures were established by extensive spectroscopic methods and comparison of their data with those of the related known compounds. All the isolates possessed a central 2-aminoimidazole ring, substituted by one or two functionalized benzyl groups in some combination of the C4 and C5 positions. The cytotoxicities against selected HL-60, HeLa, A549 and K562 tumor cell lines and anti-H1N1 (Influenza a virus (IAV)) activity for the isolates were evaluated. Compounds **1** and **2** exhibited cytotoxicities against the K562 cell line with IC_50_ values of 11.3 and 9.4 μM, respectively. Compound **5** exhibited weak anti-H1N1 (influenza a virus, IAV) activity with an inhibition ratio of 33%.

## 1. Introduction

**Figure 1:**
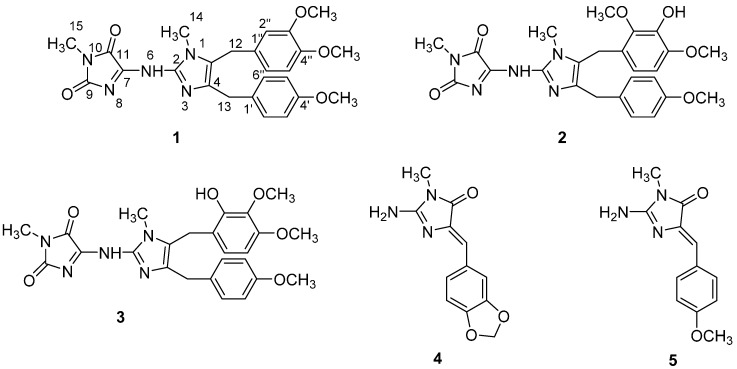
Structures of compounds **1**–**5**.

Marine sponges belonging to the Class Calcarea have been studied since the 1980s and have yielded a large number of bioactive alkaloids containing an imidazole heterocycle typically substituted with two benzylic fragments at the C-4, C-5, or N-3 positions and at various oxidation states. In some cases the 2-amino moiety is further substituted with a hydantoin or a functionalized hydantoin derivative [1]. It was reported that this kind of alkaloid showed cytotoxic [2,3,4], antimicrobial [5,6], and antifungal [7] properties, as well as leukotriene B4 receptor [8] and epidermal growth factor (EGF) receptor [9] antagonist activities. To discover new bioactive 2-aminoimidazole alkaloids, the marine sponge *Pericharax heteroraphis* (genus *Pericharax* family Leucettidae) drew our attention, as the UV characteristics of MeOH extracts showed the existence of 2-aminoimidazole alkaloids; in addition, its extracts were reported to have broad activity on cancer cell lines [10] and showed significant AChE-inhibitory activity [11]. In 2007, Ali *et al.* reported the isolation and antimicrobial activity of three 2-aminoimidazole alkaloids from *Pericharax heteroraphis* which is the only report about the chemical investigation of *Pericharax heteroraphis* [6]. Based on the above evidence, we aimed to investigate new cytotoxic compounds from *Pericharax heteroraphis.* The current sample was collected from the Yongxing Islands area of Hainan Province in the South China Sea. Combined HPLC analysis and bioassay-guided quick isolation of the imidazole alkaloid–rich portion of the MeOH extract of *Pericharax heteroraphis* yielded a new 2-aminoimidazole alkaloid (**1**), along with four known analogs (**2**–**5**) (Figure 1). Their cytotoxicities against four selected tumor cell lines and anti-H1N1 IAV (Influenza a virus) activities were evaluated. Herein, we describe the isolation, structural elucidation, and the cytotoxic and anti-IAV activities of the isolates.

## 2. Results and Discussion

### 2.1. Structure Elucidation

**Figure 2:**
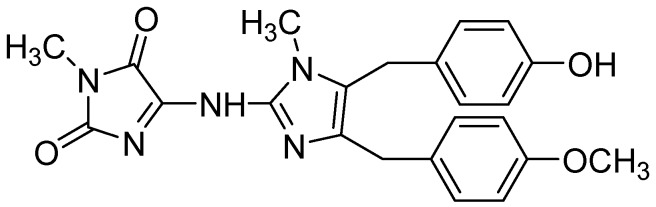
Structure of naamidine A.

**Figure 3:**
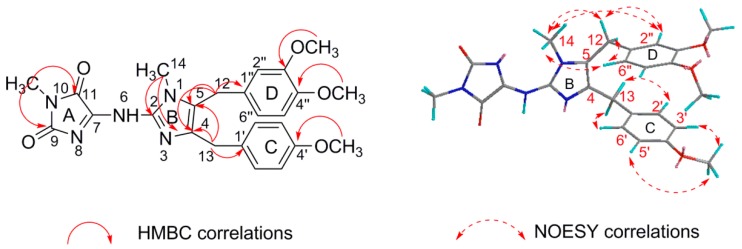
Selected key HMBC and NOESY correlations of compound **1**.

Naamidine J (**1**) was isolated as a yellow amorphous solid with a molecular formula of C_25_H_28_N_5_O_5_ established by HR-ESI-MS, which showed a pseudo-molecular-ion peak at *m*/*z* 478.2091 ([M + H]^+^ C_25_H_28_N_5_O_5_^+^; cacld. 478.2085) (see Figure S2), requiring 15 degrees of unsaturation. Its ^13^C-NMR and DEPT spectra data exhibited a total of 25 resonances for five methyl, two methylene, and seven methine groups and eleven quarternary carbons. These data also revealed the presence of 11 double bonds (7 × CC; 2 × CN; 2 × CO); the ^1^H-NMR spectrum contained three singlets at δ_H_ 3.77 (3H, s, 4′-OCH_3_), 3.84 (3H, s, 3′′-OCH_3_), 3.67 (3H, s, 4′′-OCH_3_) for three methoxy groups, and two singlets at δ_H_ 3.49 (3H, s, 14-NCH_3_), 3.17 (3H, s, 15-NCH_3_) indicating two *N*-methyl groups. In addition, the ^1^H-NMR spectrum of **1** showed a singlet aromatic signal at δ_H_ 6.42 (1H, s, H-2′′), four doublet aromatic signals at δ_H_ 7.13 (2H, d, *J* = 8.4 Hz, H-2′, 6′), 6.80 (2H, d, *J* = 8.4 Hz, H-3′, 5′), 6.74 (1H, d, *J* = 8.1 Hz, H-5′′), 6.54 (1H, d, *J* = 8.1 Hz, H-6′′) consistent with the presence of two aromatic rings: one of them 1,3,4-substituted and the other 1,4-substituted (see Figures S3–S6). The NMR data strongly suggested compound **1** to be a 2-aminoimidazole alkaloid, almost identical to those of naamidine A [12] (Figure 2) except for the 1,3,4-substituted aromatic ring (Table 1). The 1,3,4-substituted aromatic ring (ring D) were readily indicated by HMBC cross-peaks, between 3′′-OMe (δ_H_ 3.84) and C-3′′ (δ_C_ 148.1), and between 4′′-OMe (δ_H_ 3.67) and C-4′′ (δ_C_ 149.5) (Table 2) (see Figure S8). Since both H_2_-12 and H_2_-13 correlated to C-4 and C-5, the positions of the two functionalized benzyl groups were secured by NOESY correlations between 14-NCH_3_ (δ_H_ 3.49) and H_2_-12, H-2′′, H-6′′ (Figure 3) [4,12] (see Figures S9–S11). Therefore, the structure of **1** was established.

Four known analogs (Figure 1), naamidine H (**2**) [4], pyronaamidine (**3**) [13], leucettaamine B (**4**) [14], leucettamine C (**5**) [14], were also isolated and identified by comparison of their spectroscopic data with those reported in the literature.

**Table 1:** The ^1^H- and ^13^C-NMR data ^a^ for **1** ^b^ and naamidine A ^c^.

Position	1			Naamidine A		
	δ_H_, mult. (*J* in Hz)	δ_C_		δ_H_, mult. (*J* in Hz)	δ_C_	
1	-	-		-	-	
2	-	146.6	(s)	-	145.7	(s)
3	-	-		-	-	
4	-	127.0	(s)	-	133.3	(s)
5	-	129.7	(s)	-	126.9	(s)
6	-	-		-	-	
7	-	144.7	(s)	-	148.4	(s)
8	-	-	(s)	-	-	(s)
9	-	155.5	(s)	-	157.3	(s)
10	-	-		-	-	
11	-	162.3	(s)	-	162.5	(s)
12	3.91 (s)	29.2	(t)	3.91 (s)	27.7	(t)
13	3.89 (s)	32.3	(t)	3.93 (s)	30.8	(t)
14	3.49 (s)	30.1	(q)	3.39 (s)	29.6	(q)
15	3.17 (s)	24. 8	(q)	2.95 (s)	24.4	(q)
1′	-	131.7	(s)	-	131.9	(s)
2′ 6′	7.13 (d *J* = 8.4 Hz)	129.5	(d)	7.15 (d *J* = 8.6 Hz)	129.5	(d)
3′ 5′	6.80 (d *J* = 8.4 Hz)	114.2	(d)	6.81 (d *J* = 8.6 Hz)	113.9	(d)
4′	-	158.3	(s)	-	157.8	(s)
1′′	-	127.0	(s)	-	127.9	(s)
2′′	6.42 (s)	111.2	(d)	6.84 (d *J* = 8.6 Hz)	129.1	(d)
3′′	-	148.1	(s)	6.63 (d *J* = 8.6 Hz)	115.5	(d)
4′′	-	149.5	(s)	-	156.0	(s)
5′′	6.74 (d *J* = 8.1 Hz)	111.5	(d)	6.63 (d *J* = 8.6 Hz)	115.5	(d)
6′′	6.54 (d *J* = 8.1 Hz)	120.1	(d)	6.84 (d *J* = 8.6 Hz)	129.1	(d)
4′ OCH3	3.77 (s)	55.3	(q)	3.69 (s)	55.1	(q)
3′′ OCH3	3.84 (s)	55.9	(q)	-	-	(q)
4′′ OCH3	3.67 (s)	55.7	(q)	-	-	(q)

^a^ Measured at 500 MHz (^1^H) and 125 MHz (^13^C). ^b^ Measured in CDCl_3_. ^c^ Measured in CDCl_3_ + CD_3_OD.

**Table 2:** NMR data for compound **1** (Recorded in CDCl_3_) ^a^.

Position	δ_H_, mult. (*J* in Hz)	δ_C_		HMBC(H to C)	NOESY
1	-	-			
2	-	146.6	(s)		
3	-	-			
4	-	127.0	(s)		
5	-	129.7	(s)		
6	-	-			
7	-	144.7	(s)		
8	-	-	(s)		
9	-	155.5	(s)		
10	-	-			
11	-	162.3	(s)		
12	3.91 (s)	29.2	(t)	C-1′′, 2′′, 6′′, 4, 5	H-2′′, 6′′
13	3.89 (s)	32.3	(t)	C-1′, 2′, 6′, 4, 5	H- 2′, 6′
14	3.49 (s)	30.1	(q)	C-2, 5	H-12, 2′′, 6′′
15	3.17 (s)	24. 8	(q)	C-9, 11	
1′	-	131.7			
2′ 6′	7.13 (d *J* = 8.4 Hz)	129.5	(d)	C-1′, 4′, 13	
3′ 5′	6.80 (d *J* = 8.4 Hz)	114.2	(d)	C-1′, 4′	
4′	-	158.3	(s)		
1′′	-	127.0	(s)		
2′′	6.42 (s)	111.2	(d)	C-12, 6′′, 4′′	
3′′	-	148.1	(s)		
4′′	-	149.5	(s)		
5′′	6.74 (d *J* = 8.1 Hz)	111.5	(d)	C-4′′	
6′′	6.54 (d *J* = 8.1 Hz)	120.1	(d)	C-13, 5′′, 4′′	
4′ OCH3	3.77 (s)	55.3	(q)	C-4′	H-3′, 5′
3′′ OCH3	3.84 (s)	55.9	(q)	C-3′′	H-5′′
4′′ OCH3	3.67 (s)	55.7	(q)	C-4′′	H-2′′

^a 1^H- and ^13^C-NMR at 500 and 125 MHz, respectively.

### 2.2. Biological Evaluations

The cytotoxicities for all the compounds (**1**–**5**) against four tumor cell lines (human leukemia, K562; human myeloid leukemia, HL-60; human cervical carcinoma, HeLa; human lung adenocarcinoma, A549) were evaluated *in vitro* by MTT (3-(4,5-dimethylthiazol-2-yl)2,5-diphenyl-2*H*-tetrazolium bromide) and SRB (Sulforhodamine B) methods (Table 3). Compounds **1** and **2** showed modest cytotoxicities against the K562 cell line, with IC_50_ values of 11.3 and 9.4 μM, respectively. Compound **2** exhibited weak inhibitory activities against the HeLa and A549 cell lines with IC_50_ values of 21.4 and 22.4 μM, respectively (see Table S1). It is certainly noteworthy that despite the close structural similarity of compounds **1**–**5**, only compounds **1** and **2** were found to be cytotoxic.

**Table 3:** The IC_50_ values of the compounds **1**–**5** against four tumor cell lines.

Compounds	IC_50_ (μM)			
	HeLa ^a^	P388 ^b^	A549 ^a^	K562 ^b^
**1**	NA	NA	NA	11.3
**2**	21.4	NA	22.1	9.4
**3**	NA	NA	NA	NA
**4**	NA	NA	NA	NA
**5**	NA	NA	NA	NA
ADM(Adriamycin) ^c^	0.2	0.02	0.6	0.2

^a^ By SRB method; ^b^ By MTT method; ^c^ Positive control; NA inactive.

Antiviral activities of compounds **3**–**5** against H1N1 IAV were also evaluated by the cytopathic effects assays (CPE). Only compound **5** exhibited weak anti-H1N1 virus activity with an inhibition rate of 33% (Ribavirin was used as a positive control with an inhibition rate of 65%) (see Table S2). However, this is the first report of 2-aminoimidazole types of alkaloids having activity against a flu virus.

## 3. Experimental Section

### 3.1. General Methods

UV spectra were measured with a Beckman DU640 spectrophotometer (Beckman Coulter Inc., Brea, CA, USA). IR spectra were recorded on a Nicolet NEXUS 470 spectrophotometer (International Equipment Trading Ltd., Vernon Hills, IL, USA). NMR spectra were measured with Bruker 500 spectrometer (500 MHz for ^1^H and 125 MHz for ^13^C) (Bruker Daltonics Inc., Billerica, MA, USA) using TMS (tetramethylsilane) as internal standard and chemical shifts were referenced to residual non-deuterated solvent signals (CDCl_3_: δ_H_ 7.26 ppm, δ_C_ 77.16 ppm). The melting points uncorrected were measured on an X-4 micro-scopic melting point apparatus (Shanghai Instrument Physical Optics Instrument Co. Ltd., Shanghai, China). HR-ESI-MS data were obtained on a Micromass Q-Tof Ultima GLOBAL GAA076 LC mass spectrometer on a Thermo Scientific LTQ orbitrap XL mass spectrometer (Thermo Fisher Scientific Inc., Waltham, MA, USA). HPLC isolation was achieved on a Waters 2695 and Agilent 1100 instruments using semi-preparative HPLC columns (YMC-packed C18 and C8, 5 μm, 250 × 10 mm) (YMC Co. Ltd., Kyoto, Japan). Medium pressure liquid chromatograph (MPLC) was performed on a Bonna Agela LC-10F instrument using ODS (octa decylsilyl silicion) column (ODS, 50 μm, 310 × 15 mm) (Bonna-Agela Technologies Inc., Tianjin, China). Silica-gel (200–300 mesh, 300–400 mesh, Qingdao Marine Chemical Factory, Qingdao, Shandong, China) and ODS silica-gel (50 μm, Merck, Darmstadt, Germany) were used for column chromatography (CC). TLC was carried out with glass precoated silica gel GF_254_ plates (Qingdao Marine Chemical Factory, Qingdao, Shandong, China). Spots were visualized under UV light or by spraying with 10% H_2_SO_4_ in EtOH–H_2_O (95:5, *v*/*v*) followed by heating.

### 3.2. Animal Material

The marine sponge *Pericharax heteroraphis* was collected from the South Sea (Yongxing Islands area) at a depth of 12 m (16°55′32′′ N, 112°20′32′′ E) (see Figure S1), and was frozen immediately after collection. The specimen was identified by Dr. Nicole J. de Voogd (National Museum of Natural History, Leiden, the Netherlands). The voucher specimen (NO. XS 2012-28) was deposited at State Key Laboratory of Marine Drugs, Ocean University of China, Qingdao, Shandong, China.

### 3.3. Extraction and Isolation

**Figure 4:**
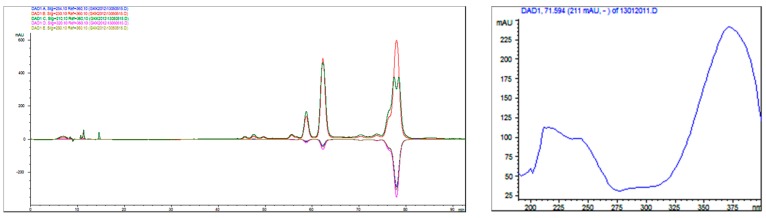
The HPLC and UV chromatograms of 2-aminoimidazole alkaloids.

The frozen sample of *Pericharax heteroraphis* (2.8 kg, wet weight) was homogenized and then extracted with MeOH three times (5 L × 3, each, three days) at room temperature, and the solution was evaporated in vacuum to yield a crude extract (36.2 g) which was subjected to column chromatography (CC) on silica gel using petroleum ether/acetone (from 100:1 to 1:2, *v*/*v*) as eluent. In the process of eluting, many yellow substances (6.7 g) were obtained and analyzed on HPLC. According to the literature and UV characteristic (Figure 4), the yellow substances could be preliminary ascertained as imidazole alkaloids thus for the further separation. Part of yellow substances (1.3 g) were subjected to medium pressure liquid chromatograph (MPLC) eluting with a gradient increasing MeOH in H_2_O to afford three main peaks (P. 1–P. 3) which were collected separately. P. 1 (12.3 mg) showed one peak on C18 and C8 column at several modified gradients but was not pure in the ^1^H-NMR spectrum thus for further purified on chiral column (CHIRALPAK^®^IC: 5μm; 100% MeOH; 0.7 mL/min) to yield compounds **4** (3.5 mg) and **5** (4.3 mg). P. 2 (40 mg) was further separated by HPLC (YMC-Pack ODS C_8_; 65% CH_3_CN in H_2_O with 1‰ HCOOH; 1.5 mL/min) to yield compound **3** (30 mg). P. 3 (24 mg) was further purified by HPLC (ODS. C8; 64% CH_3_CN in H_2_O) to yield compounds **1** (5.3 mg) and **2** (8 mg).

Compound **1**: 4-{[5-(3,4-dimethoxybenzyl)-4-(4-methoxybenzyl)-1-methyl-1*H*-imidazol-2-yl]amino}-1-methyl-1*H*-imidazole-2,5-dione. Yellow amorphous solid; melting point (m.p.): 102–104 °C; UV (MeOH): λ_max_ (log ε) 225 (4.27), 275 (3.77), 392 (3.97) nm; IR (KBr): 3427, 2960, 2873, 1731, 1710, 1377, 1250, 1068, 1025 cm^−1^; ^1^H and ^13^C-NMR: see Table 1; HR-ESI-MS: *m/z* 478.2091 [M + H]^+^ (calcd. for C_25_H_28_O_5_N_5_^+^, 478.2085); 500.1906 [M + Na]^+^ (calcd. for C_25_H_27_NaO_5_N_5_^+^, 500.1904).

### 3.4. Cytotoxic Assay

*In vitro* cytotoxicities were determined by MTT [3-(4,5-dimethylthiazol-2-yl)-2, 5-diphenyltetrazolium bromide]colorimetric assay against K562 (human leukemia cells) and HL-60 (human myeloid leukemia cells), SRB (Sulforhodamine B) assay against HeLa (human cervical carcinoma cells) and A549 (human lung adenocarcinoma cells). All the cell lines were purchased from Shanghai Institute of Cell Biology (Shanghai, China). Cytotoxic data (Table 3) for compounds **1**–**5** were obtained on the basis of five concentrations with three replications. Adriamycin (doxorubicin, ADM) was used as a positive control, and IC_50_ values > 50 μM were considered to be inactive in cytotoxic assays.

In the MTT assay [15], the cells were cultured in RPMI-1640 supplemented with 10% FBS under a humidified atmosphere of 5% CO_2_ and 95% air at 37 °C. Those cell suspensions (200 μL) at a density of 5 × 10^4^ cell mL^−1^ were plated in 96-well microtiter plates and incubated for 24 h at the above conditions. The test compound solution (2 μL in DMSO) at different concentrations in triplicate was added to each well and was further incubated for 72 h under the same conditions. 20 μL of the MTT solution (5 mg/mL in IPMI-1640 medium) was then added to each well and incubated for 4 h. The old medium containing MTT (150 μL) was then gently replaced by DMSO and vibrated to dissolve any formazan [1-(4-Iodophenyl)-5-(4-nitrophenyl)-3-phenylformazan] crystals formed. The optical density of the solution was measured on a Spectra Max Plus plate reader at 570 nm. The IC_50_ value of each compound was calculated by Reed and Muench’s method.

In the SRB assay [16], 200 μL of the cell suspensions were plated in 96-well plates at a density of 2 × 10^5^ cell mL^−1^. Then 2 μL of the test solutions (in MeOH) was added to each well, and the culture was further incubated for 24 h. The cells were fixed with 12% trichloroacetic acid, and the cell layer was stained with 0.4% SRB. The absorbance of the SRB solution was measured at 515 nm. Dose-response curves were generated, and the IC_50_ values (the concentration of compound required to inhibit cell proliferation by 50%) were calculated from the linear portion of log dose-response curves.

### 3.5. Anti-H1N1 Virus Assay

The antiviral activity against H1N1 was evaluated by the cytopathic effects assays (CPE) [17]. Confluent MDCK (Madin-Daby canine kidney) cell monolayers were firstly incubated with influenza virus (A/Puerto Rico/8/34 (H1N1), PR/8) at 37 °C for 1 h. After removing the virus dilution, cells were maintained in infecting media (RPMI 1640, 4 μg/mL of trypsin) containing different concentrations of test compounds at 37 °C. After 48 h incubation at 37 °C, cells were fixed with 100 μL of 4% formaldehyde for 20 min at room temperature. After removal of the formaldehyde, the cells were stained with 0.1% crystal violet for 30 min. The plates were washed and dried, and the intensity of crystal violet staining for each well was measured in a microplate reader (Bio-Rad, Hercules, CA, USA) at 570 nm. The IC_50_ was calculated as the compound concentration required inhibiting CPE production at 48 h post-infection by 50%. Ribavirin (LuKang Cisen, Jining, China) was used as positive control, and compounds with an inhibition rate of >70%, >50%, and <30% at 50 μg/mL were respectively regarded as having strong, moderate, and weak activities.

## 4. Conclusions

The present work offered one new member of 2-aminoimidazole alkaloids and the first isolation of compounds **1**, **2**, **3** and **5** from the genus *Pericharax*, and firstly reported anti-H1N1 virus activities of this type of alkaloid. Compounds **1** and **2** showed cytotoxic activity against the K562 cell line, suggesting that they might have the potential to be developed as antitumor agents. It was reported that the extract of *Pericharax heteroraphis* showed significant anti-AChE-inhibitory activity, and continuing investigation of the anti-AChE-inhibitory activity of the isolates is worthwhile.

## Supplementary Materials

Supplementary materials can be accessed at: http://www.mdpi.com/1420-3049/21/2/150/s1.
