# Supplementary material for: Imidazole Alkaloids from the South China Sea Sponge Pericharax heteroraphis and Their Cytotoxic and Antiviral Activities
Source: Molecules. 2016 Jan 26;21(2):150. doi: 10.3390/molecules21020150 (PMC6274532; doi:10.3390/molecules21020150)
Supplement: Supplementary file 1 [file molecules-21-00150-s001.pdf]

Supplementary Information: Imidazole Alkaloids from the South China Sea Sponge *Pericharax heteroraphis* and their Cytotoxic and Antiviral Activities

Kai-Kai Gong, Xu-Li Tang, Yi-Sheng Liu, Ping-Lin Li and Guo-Qiang Li

Table1 S1. Inhibition rates of compounds **1–5** in preliminary cytotoxicity test.

Table1 S2. Inhibition rates of compounds with anti–H1N1 virus activities in preliminary test.

Figure S1. Animal material.

Figure S2. The positive HRESIMS spectrum of compound (**1**).

Figure S3. The ^{1}H-NMR (500 MHz, $CDCl_3$) spectrum of compound (**1**).

Figure S4. The amplificatory ^{1}H NMR (500 MHz, $CDCl_3$) spectrum of compound (**1**).

Figure S5. The ^{13}C-NMR (125 MHz, $CDCl_3$) spectrum of compound (**1**).

Figure S6. DEPT (125 MHz, $CDCl_3$) spectrum of compound (**1**).

Figure S7. HMQC spectrum of compound (**1**).

Figure S8. HMBC spectrum of compound (**1**).

Figure S9. NOESY spectrum of compound (**1**).

Figure S10. The amplificatory NOESY spectrum of compound (**1**).

Figure S11. The amplificatory NOESY spectrum of compound (**1**).

Table S1. Inhibition rates of compounds **1–5** in preliminary cytotoxicity test.

	K562		HL-60		HeLa		A549	
Compounds	Inhibition Ratio (%)	OD Value	Inhibition Ratio (%)	OD Value	Inhibition Ratio (%)	OD Value	Inhibition Ratio (%)	OD Value
Adramycin (1 μM)	56.67	0.31 ± 0.00	93.96	0.11 ± 0.00	69.58	0.49 ± 0.01	57.41	0.46 ± 0.02
**1** (50 μM)	92.92	0.13 ± 0.00	94.16	0.10 ± 0.00	96.46	0.06 ± 0.00	93.75	0.07 ± 0.01
**2** (50 μM)	89.74	0.15 ± 0.01	15.36	1.51 ± 0.04	96.19	0.06 ± 0.00	93.75	0.07 ± 0.01
**3** (50 μM)	−1.42	0.13 ± 0.00	13.09	1.55 ± 0.01	-3.83	1.67 ± 0.02	8.52	0.98 ± 0.05
**4** (50 μM)	13.02	0.36 ± 0.00	8.74	1.63 ± 0.05	2.11	1.58 ± 0.01	9.88	0.97 ± 0.01
**5** (50 μM)	0.14	0.14 ± 0.00	13.94	1.54 ± 0.03	5.97	1.51 ± 0.04	2.67	1.04 ± 0.04

Table S2. Inhibition rates of compounds with anti–H1N1 virus activities in preliminary test.

compounds	Concentration (μg/mL)	Inhibition Ratio (%)
Ribavirin	50	65
**3**	50	20.8
**4**	50	0.7
**5**	50	33.0

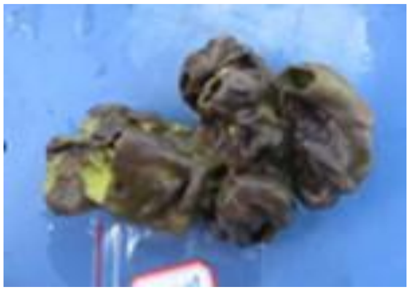

Pericharax heteroraphis

Figure S1. Animal material: The marine sponge *Pericharax heteroraphis* was collected from the South Sea (Yongxing Islands area) at a depth of 12 m (16°55' 32'' N，112°20' 32'' E), and was frozen immediately after collection. The specimen was identified by Dr. Nicole J. de Voogd (National Museum of Natural History, Leiden, The Netherlands). The voucher specimen (NO. XS 2012-28) was deposited at State Key Laboratory of Marine Drugs, Ocean University of China, P. R. Qingdao, Shandong, China.

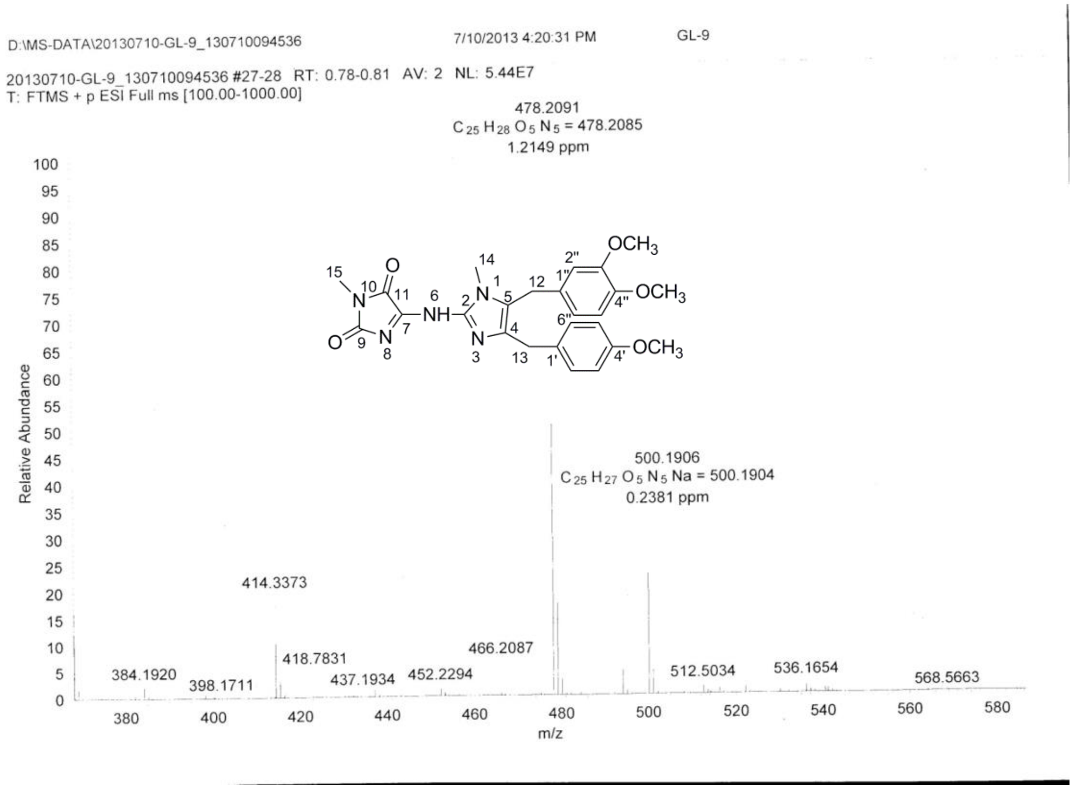

Figure S2. The positive HRESIMS spectrum of compound (**1**).

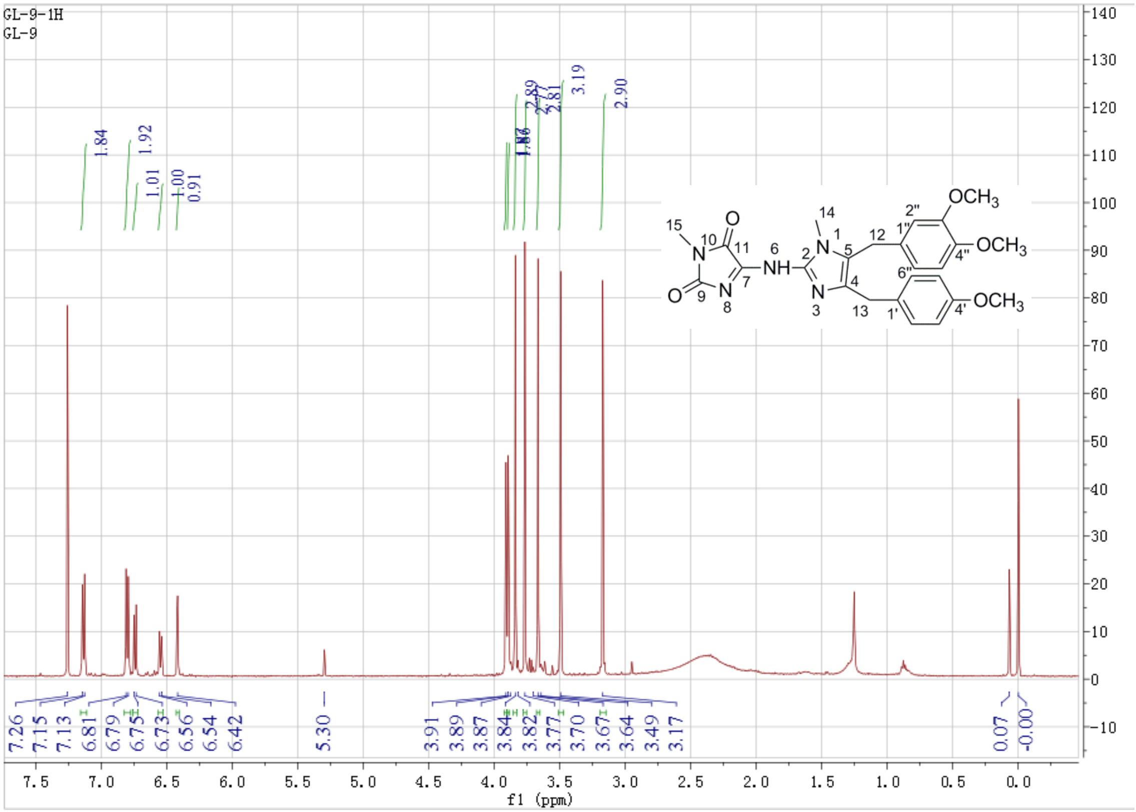

Figure S3. The^{1}H-NMR (500 MHz, $CDCl_3$) spectrum of compound (**1**).

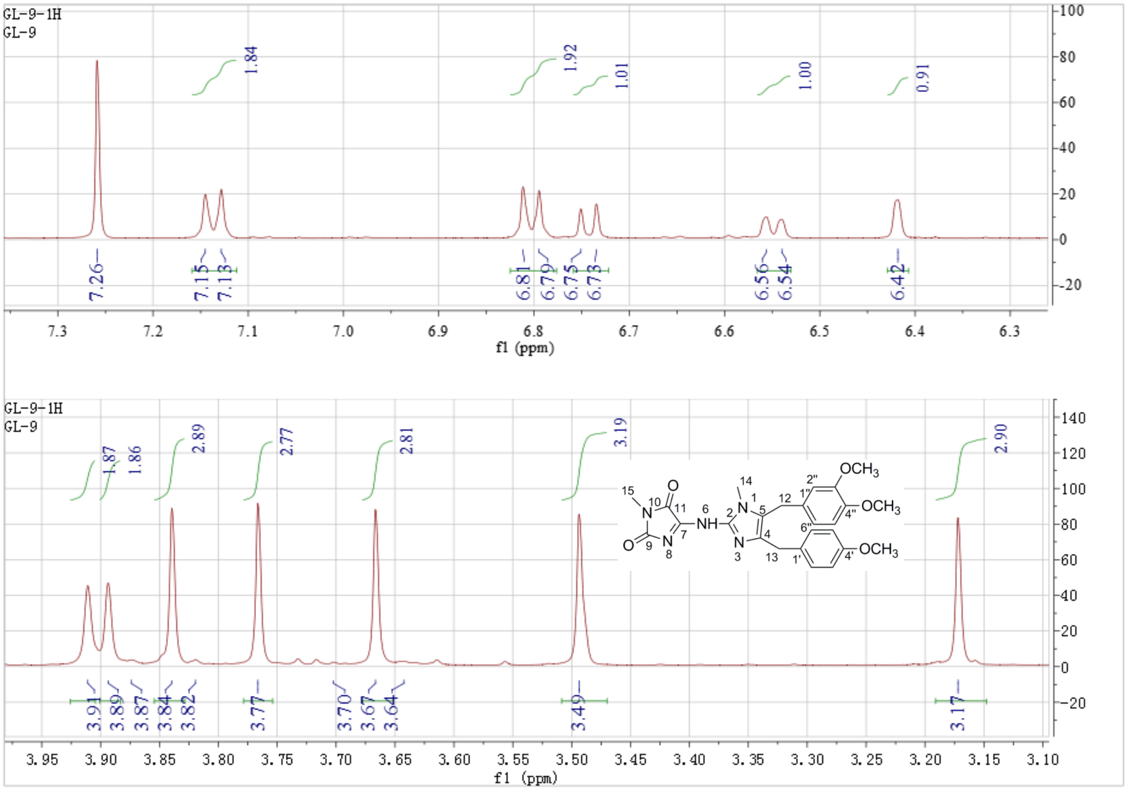

Figure S4. The amplificatory ^{1}H NMR (500 MHz, $CDCl_3$) spectrum of compound (**1**).

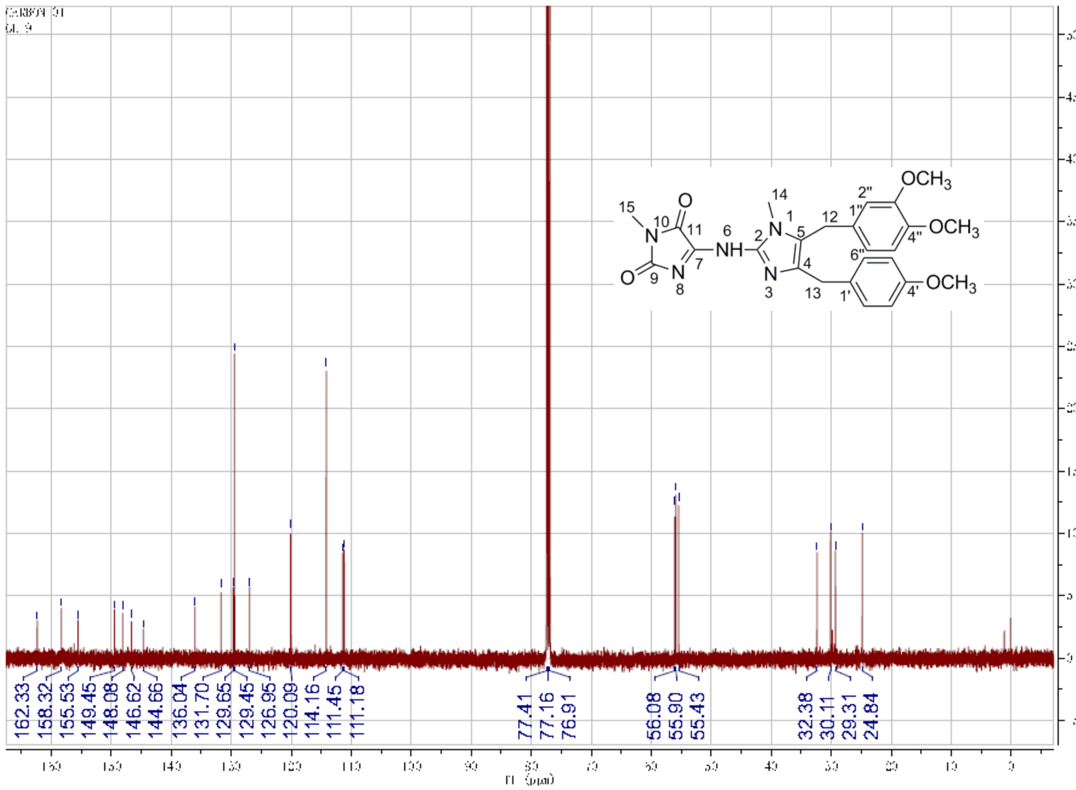

Figure S5. The ^{13}C-NMR (125 MHz, $CDCl_3$) spectrum of compound (**1**).

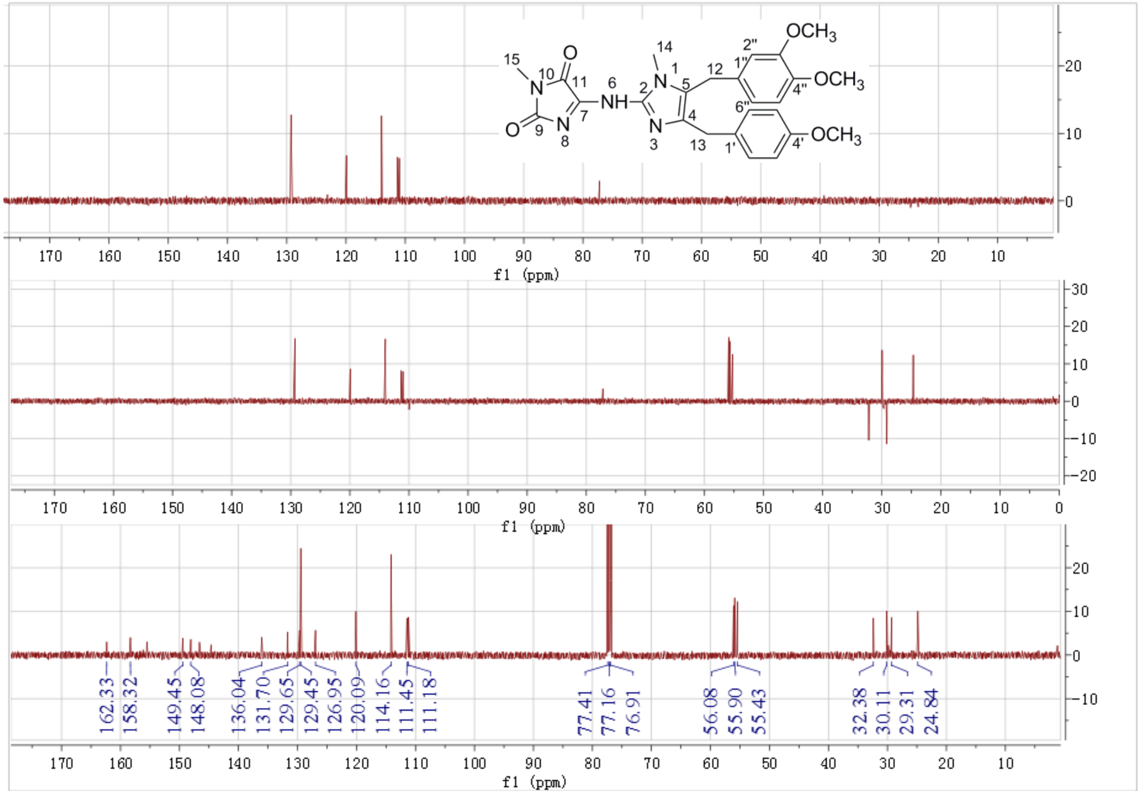

Figure S6. DEPT (125 MHz, $CDCl_3$) spectrum of compound (**1**).

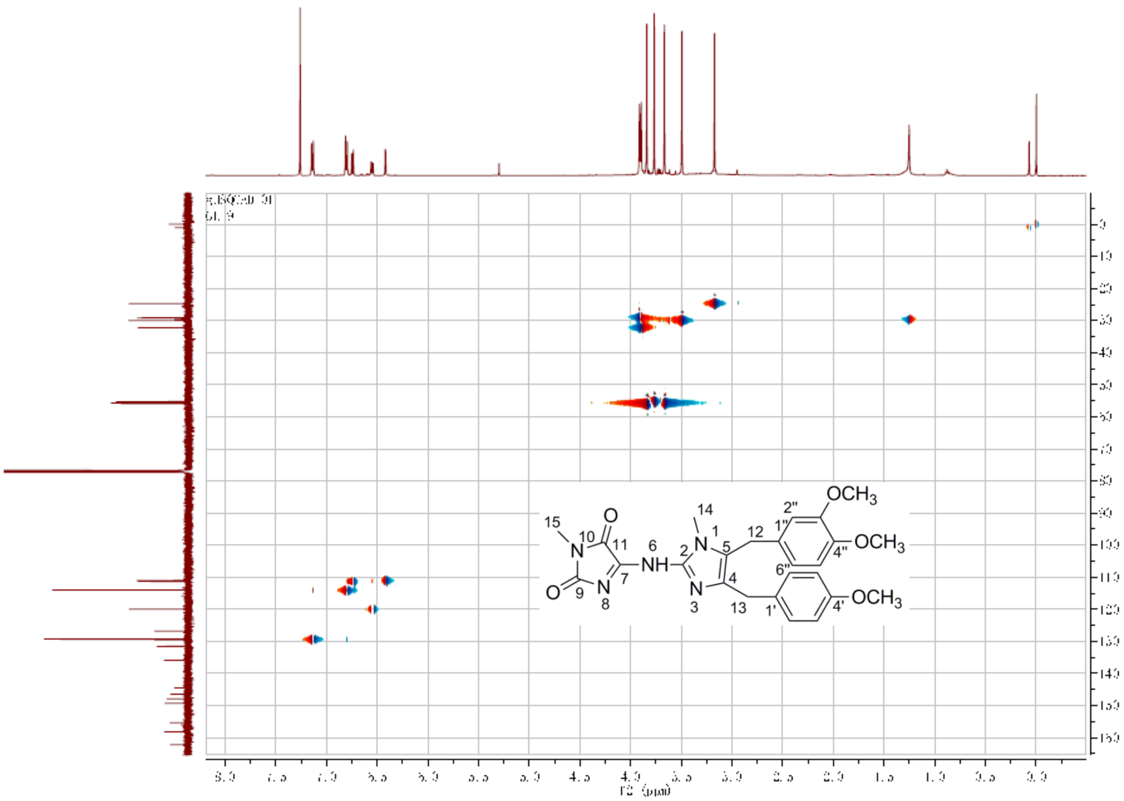

Figure S7. HMQC spectrum of compound (**1**).

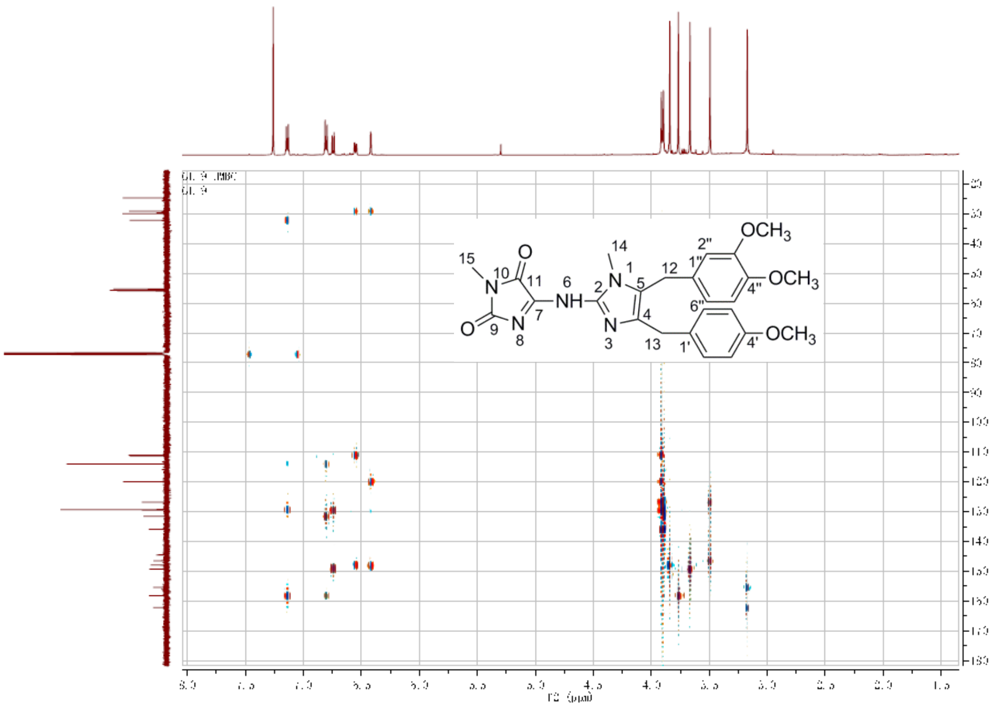

Figure S8. HMBC spectrum of compound (**1**).

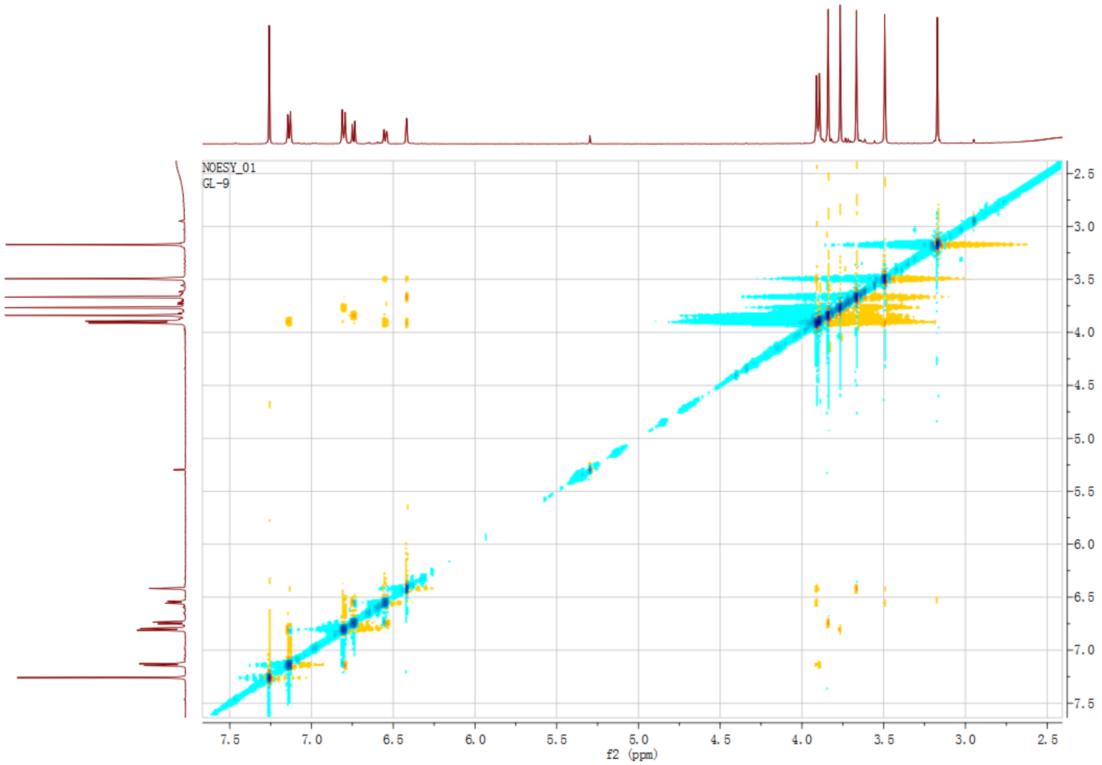

Figure S9. NOESY spectrum of compound (**1**).

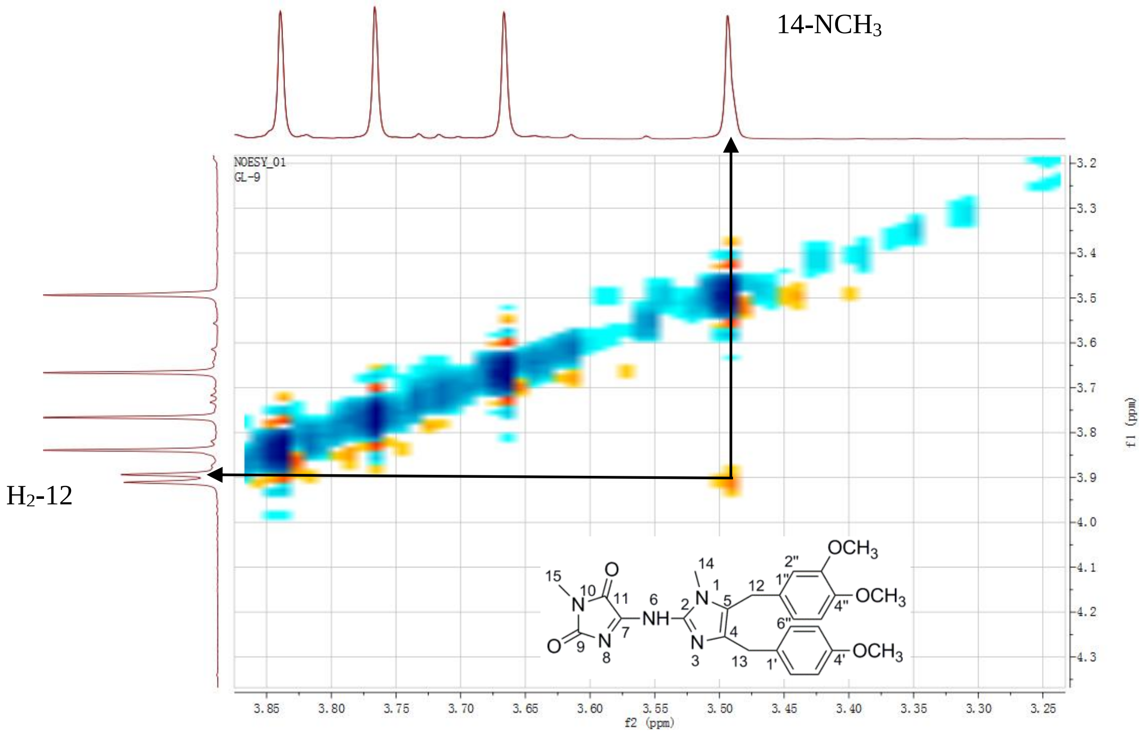

Figure S10. The amplificatory NOESY spectrum of compound (**1**).

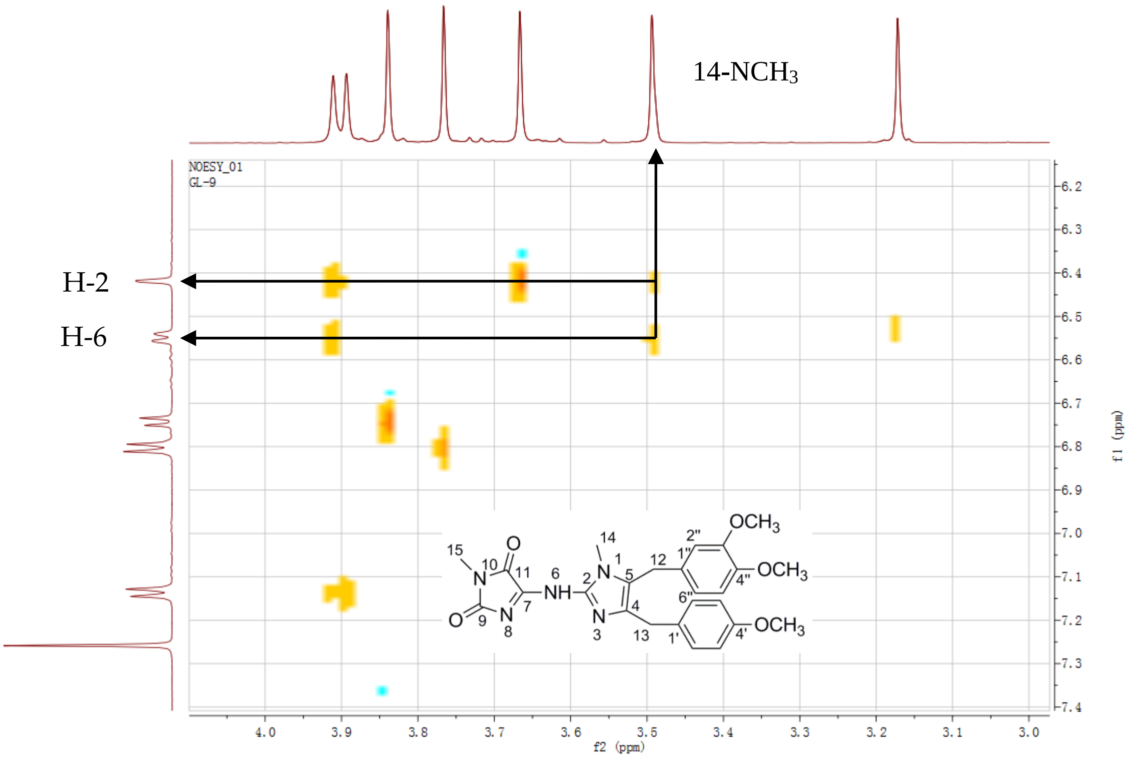

Figure S11. The amplificatory NOESY spectrum of compound (**1**).
